# MRI-based radiomics of rectal cancer: preoperative assessment of the pathological features

**DOI:** 10.1186/s12880-019-0392-7

**Published:** 2019-11-12

**Authors:** Xiaolu Ma, Fu Shen, Yan Jia, Yuwei Xia, Qihua Li, Jianping Lu

**Affiliations:** 1 Department of Radiology, Changhai Hospital of Shanghai, Shanghai, China; 2 Huiying Medical Technology Co., Ltd, Beijing, China

**Keywords:** Histological grade, Magnetic resonance imaging, N stage, Radiomics, Rectal cancer, T stage

## Abstract

**Background:**

This study aimed to evaluate the significance of MRI-based radiomics model derived from high-resolution T2-weighted images (T2WIs) in predicting tumor pathological features of rectal cancer.

**Methods:**

A total of 152 patients with rectal cancer who underwent surgery without any neoadjuvant therapy between March 2017 and September 2018 were included retrospectively. The patients were scanned using a 3-T magnetic resonance imaging, and high-resolution T2WIs were obtained. Lesions were delineated, and 1029 radiomics features were extracted. Least absolute shrinkage and selection operator was used to select features, and multilayer perceptron (MLP), logistic regression (LR), support vector machine (SVM), decision tree (DT), random forest (RF), and K-nearest neighbor (KNN) were trained using fivefold cross-validation to build a prediction model. The diagnostic performance of the prediction models was assessed using the receiver operating characteristic curves.

**Results:**

A total of 1029 features were extracted, and 15, 11, and 11 features were selected to predict the degree of differentiation, T stage, and N stage, respectively. The best performance of the radiomics model for the degree of differentiation, T stage, and N stage was obtained by SVM [area under the curve (AUC), 0.862; 95% confidence interval (CI), 0.750–0.967; sensitivity, 83.3%; specificity, 85.0%], MLP (AUC, 0.809; 95% CI, 0.690–0.905; sensitivity, 76.2%; specificity, 74.1%), and RF (AUC, 0.746; 95% CI, 0.622-0.872; sensitivity, 79.3%; specificity, 72.2%).

**Conclusion:**

This study demonstrated that the high-resolution T2WI–based radiomics model could serve as pretreatment biomarkers in predicting pathological features of rectal cancer.

## Background

Colorectal cancer (CRC) is the third most common malignant tumor worldwide [1]. According to the latest data, reported by the Cancer Statistics of China in 2015, CRC ranks fifth in morbidity and mortality [2]. Among all the patients with CRC, rectal cancer accounts for 30–35%, which are generally adenocarcinomas. The optimal therapy program selection is a multifarious course for patients with rectal cancer [3, 4], and an accurate preoperative stage is an essential step for guiding treatment decisions, including surgery or neoadjuvant chemoradiotherapy (nCRT). Surgical excision is regarded as the standard treatment strategy for early rectal cancer (T1–2 and N0), and the treatment for locally advanced (T3–4 and/or N1) rectal cancer is nCRT followed by total mesorectal excision surgery [3]. Generally, the pathological type, degree of differentiation, depth of infiltration, and presence or absence of regional lymph node metastasis reflect the degree of tumor invasiveness and predict the prognosis of a tumor [3]. Therefore, a deeper understanding of tumor pathological features has a critical value in formulating the clinical treatment plan and predicting the prognosis. Moreover, high-resolution magnetic resonance imaging (MRI) has a pivotal role in the pretreatment assessment of rectal cancer because the high-resolution T2-weighted images (T2WIs) offer better diagnostic performance in the staging of rectal cancer [3].

Recently, radiomics analysis was developed and validated as an advanced tool in assessing tumor heterogeneity. Radiomics is a noninvasive method that involves high-quality image acquisition, VOIs segmentation, high-throughput quantitative feature extraction, high-dimension feature reduction, and diagnostic, prognostic, or predictive model establishment. The radiomics model, which makes use of the medical images and clinical data, has a potential in clinical decision-making [5]. Radiomics has been used to evaluate several kinds of tumors in previous studies and is being increasingly implemented [5–9]. MRI-based radiomics model has been employed in distinguishing cancer from benign tissue and reflecting the histological characteristics of rectal cancer [10–13]. Therefore, the purpose of the present study was to investigate the significance of an MRI-based radiomics model derived from high-resolution T2WI in identifying specific pathological features of rectal cancer and build a set of prediction radiomics models.

## Methods

### Participants

This retrospective study was approved by the local institutional (Committee on Ethics of Biomedicine, Second Military Medical University) review board, and written informed consent was waived for each patient. Between March 2017 and September 2018, 182 consecutive patients with rectal lesions identified by colonoscopy with no previous treatment were involved in this study. All patients underwent rectal MRI examination and postoperative pathological test. The exclusion criteria were as follows: chemotherapy or radiotherapy before and after MRI (*n* = 20), poor image quality (*n* = 6), and distant metastases (*n* = 4). Therefore, 152 patients were included in the final analysis.

### Magnetic resonance imaging

All patients were scanned on a 3 T MRI (MAGNETOM Skyra, Siemens Healthcare, Erlangen, Germany) using an 18-channel pelvic phased-array coil. Every patient fasted for 4 h prior to the scan. Transversal high-resolution T2-weighted turbo spin echo images were acquired with the following parameters: TR/TE = 4000/108 ms, FOV = 180 × 180 mm^2^, matrix = 320 × 320, slice thickness = 3 mm, gap = 0 mm, acceleration factor = 3, echo train length = 16, and acquisition time = 4 min 10 s. All patients underwent surgery at a time interval of 8.9 ± 5.8 (range, 2–28) days after the MRI examination.

### Pathological evaluation

The tissue sections were subjected to hematoxylin and eosin staining. All lymph nodes in the mesorectum were retrieved from the surgical specimens to ensure that at least 12 lymph nodes per patient were collected. The final histopathological reports detailed the tumor TN staging, histological grade, and circumferential resection margin (CRM). All TN statuses were determined according to the American Joint Committee on Cancer staging system, eighth edition [14, 15]. The patients were divided into two groups according to different pathological criteria. Histological grade: high-to-moderate and poor differentiation; T stage: T1–2 and T3–4 stages; and N stage: N0 and N1–2 stages.

### Feature selection

**Fig. 1:**
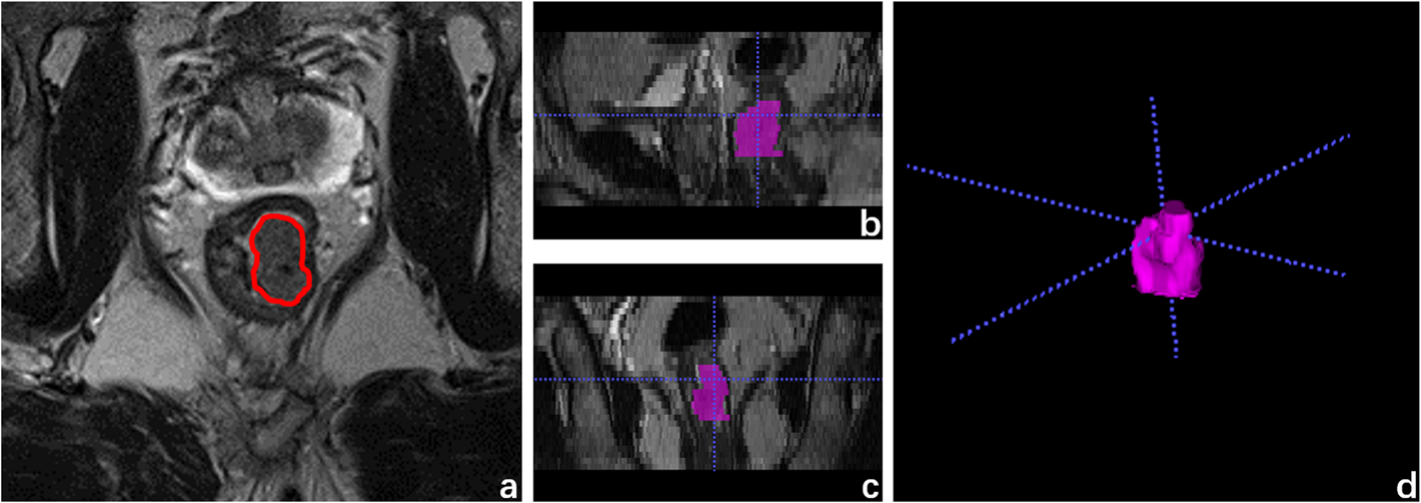
Example image for rectal cancer contouring. **a** The outline of ROI on one slice of axial T2-weighted MR image. **b** Sagittal reconstruction. **c** Coronal reconstruction. **d** Volume rendering

The radiomics features were extracted from the VOIs as confirmed by a radiologist (with 8 years of experience in radiology) on high-resolution T2WI using a radiomics analysis platform [Radcloud, Huiying Medical Technology (Beijing, China) Co., Ltd.] (Fig. 1). 1029 high-throughput data features based on feature classes and filter classes were automatically extracted from the platform. The platform feature extraction is based on the “pyradiomics” package in Python (version 2.1.2, https://pyradiomics.readthedocs.io/).

To minimize the MRI intensity variations, we normalized the intensity of the image using the following formula (where *x* indicates the original intensity; *f*(*x*) indicates the normalized intensity; *μ* refers to the mean value; *σ* indicates the variance; *s* is an optional scaling, by default, it is set to 1).

$$ f(x)=\frac{s\left(x-{\mu}_x\right)}{\sigma_x} $$

First, to guarantee image feature robustness, the basis of an intraclass correlation of 0.6 was set for test–retest analysis. Then, the robust features were selected by the least absolute shrinkage and selection operator (LASSO) method to best predict the classification performance. In the LASSO method, leave-one-out cross-validation was used to select the optimal regularization parameter alpha, as the average of mean square error of each patient was the smallest. With the optimal alpha, features having nonzero coefficient in LASSO were reserved.

### Prediction model analysis

The machine learning is based on the “scikit-learn” package in Python (version 0.21.3, https://scikit-learn.org/stable/). The original collection was divided into a training set (70%) and a test set (30%) randomly. Moreover, to lower the imbalance impact of samples distribution of the degree of histological grade and N stage, the synthetic minority oversampling technique algorithm was used in the training set. The multilayer perceptron (MLP), logistic regression (LR), support vector machine (SVM), decision tree (DT), random forest (RF), and K-nearest neighbor (KNN) classifiers were trained (the parameters of the six classifiers are shown in Table 1) using fivefold cross-validation to build a prediction model. Moreover, the independent test set was used to test the performance of the model. The experiment used the mean model as the final model for the test set. The performance of models for the statistically significant pathological features was assessed using sensitivity, specificity, and area under the receiver operating characteristic (ROC) curve (AUC). *P* value < 0.05 was considered statistically significant.

**Table 1:** Supplemental data (parameters)

Model	Degree of Differentiation	T stage	N stage
MLP	hidden_layer_sizes = (30)	hidden_layer_sizes = (30)	hidden_layer_sizes = (30)
LR	penalty = ‘l2’, solver = ‘liblinear’	penalty = ‘l2’, solver = ‘liblinear’	penalty = ‘l2’, solver = ‘liblinear’
SVM	kernel = ‘rbf’, probability = True	kernel = ‘Poly’, probability = True	kernel = ‘rbf’, probability = True
DT	criterion = ‘gini’	criterion = ‘gini’	criterion = ‘gini’
RF	n_estimators = 15	n_estimators = 15	n_estimators = 15
KNN	n_neighbors = 5	n_neighbors = 5	n_neighbors = 5

## Results

### Patient demographics

Among the 152 patients with rectal cancer, 94 were male and 58 were female, with a mean age of 58.9 ± 8.3 years (range 24–78). The pathological features of rectal cancer are presented in Table 2. None of them had positive CRM.

**Table 2:** Pathological characteristics of the patients

pathological characteristics	Total	Training data (70%)	Test data (30%)
	n percentile (%)	n percentile (%)	n percentile (%)
Gender			
Male	94 (61.8)	63 (59.4)	31 (67.4)
Female	58 (38.2)	43 (40.6)	15 (32.6)
Age (years)			
Mean	58.9 ± 8.3	52.3 ± 10.	58.9 ± 8.0
Range	24–78	24–77	25–78
Histological type			
Adenocarcinoma	131 (86.2)	91 (85.8)	40 (87.0)
Mucinous adenocarcinoma	15 (9.9)	11 (10.4)	4 (8.7)
Signet ring cell carcinoma	6 (3.9)	4 (3.8)	2 (4.3)
Pathologic differentiation			
High	20 (13.2)	14 (13.2)	6 (13.0)
Moderate	112 (73.7)	78 (73.6)	34 (73.9)
Poor	20 (13.2)	14 (13.2)	6 (13.0)
T stage			
T1	22 (14.5)	15 (14.2)	7 (15.2)
T2	44 (28.9)	28 (26.4)	16 (34.8)
T3	74 (48.7)	53 (50.0)	21 (45.7)
T4	12 (7.9)	10 (9.4)	2 (4.3)
N stage			
N0	94 (61.9)	67 (63.2)	27 (58.7)
T1	37 (24.3)	27 (25.5)	10 (21.7)
T2	21 (13.8)	12 (11.3)	9 (19.6)

### Diagnostic performance of radiomics

A total of 1029 features were extracted from preoperative high-resolution T2WI, can be classified into three categories as follows: I. The characteristics of intensity statistics, such as peak value, mean value, and variance, which are used to quantitatively describe the distribution of voxel intensity in MR images; II. Shape features, such as volume, surface area, and spherical value, which reflect the three-dimensional characteristics of the shape and size of the outlined area; and III. texture features, including the gray-level co-occurrence matrix, gray-level run length matrix, and gray-level size zone matrix, which can quantify the heterogeneity of the selected region. Additionally, Laplace-Gauss filtering, exponential, logarithmic, square, square root, and wavelet filters can be used to calculate image intensity and texture features. Wavelet filters used included wavelet-LHL, wavelet-LHH, wavelet-HLL, wavelet-LLH, wavelet-HLH, wavelet-HHHH, wavelet-HHL, and wavelet-LLL. Then 15, 11, and 11 characteristic features related to the degree of differentiation, T stage, and N stage, respectively, were obtained (Table 3). Radiomics features were selected for subsequent prediction model building, the cutoff value was selected according to the Youden index to determine the corresponding sensitivity and specificity. The AUC was used to assess the predictive ability of the model, and the selection results are presented in Tables 4 and 5.

**Table 3:** Radiomics features

No	Degree of differentiation	T stage	N stage
1	original_firstorder_Minimum	original_shape_Size	WaveletHLH_firstorder_Medianvalue
2	original_firstorder_Entropy	WaveletLLH_firstorder_Medianvalue	WaveletHLL_glrlm_SRLGE
3	original_shape_Compactness	WaveletLHH_firstorder_Meanvalue	WaveletHHL_firstorder_Energy
4	original_glrlm_RLV	WaveletLHH_firstorder_Uniformity	WaveletLLH_firstorder_Medianvalue
5	WaveletLLH_firstorder_Skewness	WaveletHHL_firstorder_Medianvalue	WaveletHHH_glszm_LGZE
6	WaveletLLH_firstorder_Uniformity	WaveletLLL_glszm_SZE	WaveletLLL_glrlm_LRHGE
7	WaveletHLH_firstorder_Kurtosis	WaveletLLL_glszm_ZSN	WaveletHHL_firstorder_Skewness
8	WaveletLHL_glszm_LGZE	WaveletLLL_ngtdm_Coarseness	WaveletLLL_glcm_cshad
9	WaveletLLL_glrlm_LRHGE	WaveletHLH_glcm_inf1h	WaveletLLL_glrlm_HGRE
10	WaveletHHH_glrlm_RLV	WaveletHHL_glcm_senth	WaveletHLL_ngtdm_Coarseness
11	WaveletHHH_glszm_LGZE	WaveletHHL_glrlm_LRHGE	WaveletHLL_glcm_inf1h
12	WaveletHHL_glcm_inf2h		
13	WaveletHHH_glcm_cprom		
14	WaveletHHH_glcm_corrm		
15	WaveletLHH_glrlm_GLV

**Table 4:** Training set

pathological features	model	mean AUC	std	sensitivity	specificity	Youden index
degree of differentiation	MLP	0.942	0.041	0.871	0.978	0.849
	LR	0.874	0.052	0.806	0.903	0.709
	SVM	0.871	0.037	0.806	0.892	0.698
	DT	0.892	0.040	1.0	1.0	1.0
	RF	0.983	0.020	1.0	1.0	1.0
	KNN	0.933	0.062	0.978	0.860	0.838
T stage	MLP	0.824	0.087	0.804	0.900	0.704
	LR	0.792	0.083	0.826	0.733	0.559
	SVM	0.764	0.083	0.913	0.783	0.696
	DT	0.722	0.060	1.0	1.0	1.0
	RF	0.713	0.031	1.0	0.983	0.983
	KNN	0.712	0.081	0.956	0.600	0.556
N stage	MLP	0.694	0.122	0.861	0.677	0.538
	LR	0.651	0.089	0.831	0.492	0.323
	SVM	0.684	0.143	0.831	0.738	0.569
	DT	0.713	0.060	1.0	1.0	1.0
	RF	0.794	0.100	1.0	0.954	0.954
	KNN	0.663	0.060	1.0	1.0	1.0

**Table 5:** Test set

pathological features	model	AUC	95% CI	sensitivity	specificity	Youden index
degree of differentiation	MLP	0.825	0.659–0.967	0.833	0.750	0.583
	LR	0.808	0.649–0.946	0.833	0.725	0.558
	SVM	0.862	0.750–0.967	0.833	0.850	0.683
	DT	0.854	0.700–0.963	0.833	0.875	0.708
	RF	0.858	0.735–0.964	0.833	0.750	0.583
	KNN	0.692	0.519–0.844	0.833	0.450	0.283
T stage	MLP	0.809	0.690–0.905	0.762	0.741	0.503
	LR	0.762	0.633–0.873	0.714	0.630	0.344
	SVM	0.753	0.623–0.857	0.667	0.630	0.297
	DT	0.667	0.543–0.783	0.667	0.667	0.334
	RF	0.727	0.591–0.843	0.714	0.704	0.418
	KNN	0.720	0.586–0.830	0.809	0.407	0.216
N stage	MLP	0.667	0.531–0.799	0.690	0.722	0.412
	LR	0.437	0.294–0.575	0.448	0.444	−0.108
	SVM	0.592	0.435–0.736	0.552	0.500	0.052
	DT	0.723	0.599–0.832	0.724	0.722	0.446
	RF	0.746	0.622–0.872	0.793	0.722	0.515
	KNN	0.560	0.428–0.69	0.621	0.500	0.121

**Fig. 2:**
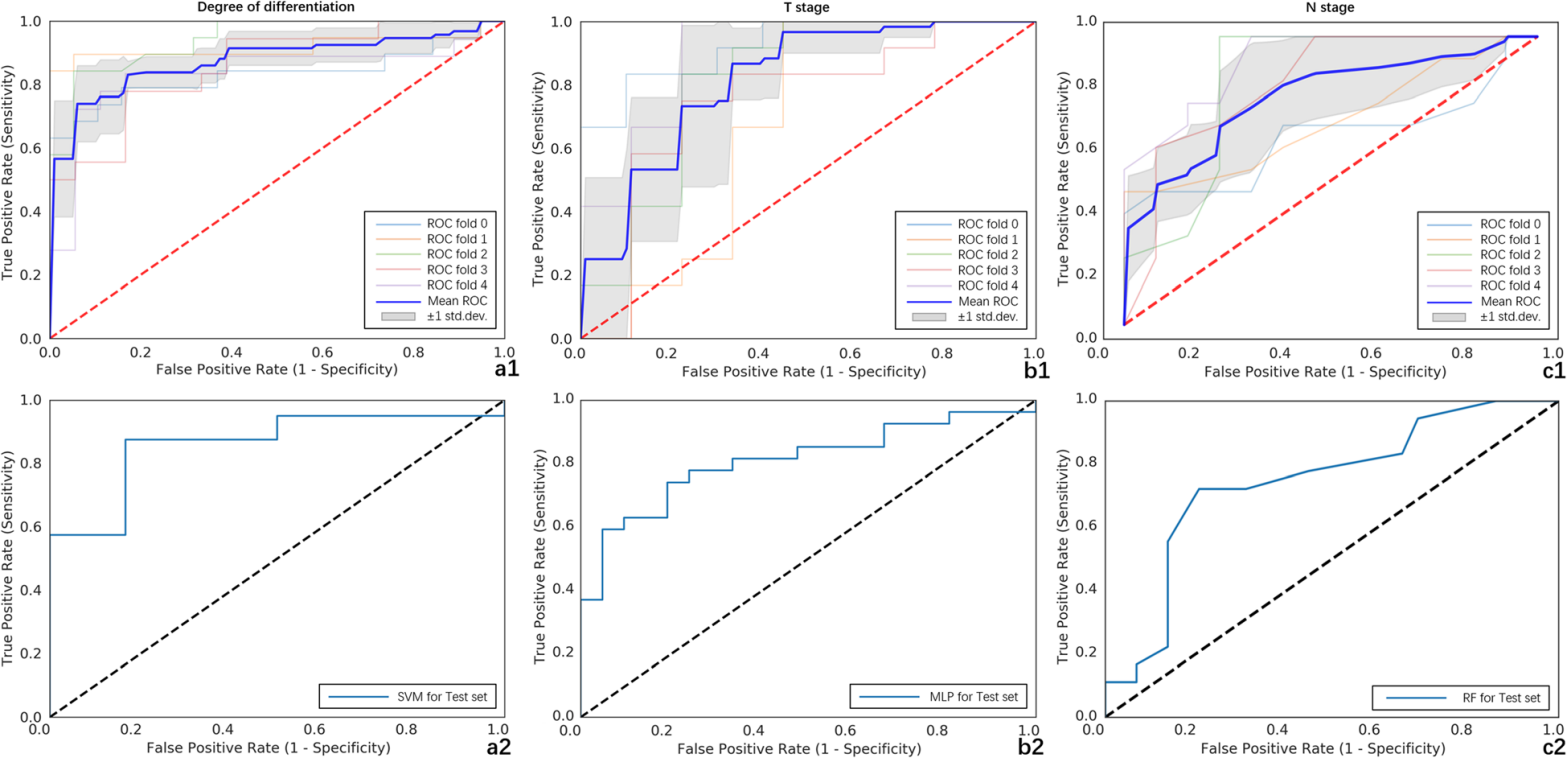
Receiver operating characteristic (ROC) curves of the prediction model for the statistically significant prognostic factors. ROC curves of SVM classifier for pathological differentiation: (**a1**) training set (AUC, 0.871; std., 0.037; sensitivity, 80.6%; specificity, 89.2%); (**a2**) test set (AUC, 0.862; 95% CI, 0.750–0.967; sensitivity, 83.3%; specificity, 85.0%). ROC curves of MLP classifier for T stage: (**b1**) training set (AUC, 0.824; std., 0.087; sensitivity, 80.4%; specificity, 90.0%); (**b2**) test set (AUC, 0.809; 95% CI, 0.690–0.905; sensitivity, 76.2%; specificity, 74.1%). ROC curves of RF classifier for N stage: (**c1**) training set (AUC, 0.794; std., 0.100; sensitivity, 100.0%; specificity, 95.4%); (**c2**) test set (AUC, 0.746; 95% CI, 0.622–0.872; sensitivity, 79.3%; specificity, 72.2%)

For the degree of differentiation, the SVM classifier provided the best discrimination capability for the prediction model with an AUC of 0.862 (95% CI, 0.750–0.967; sensitivity, 83.3%; specificity, 85.0%). As for the T stage, the MLP classifier provided the best discrimination capability with an AUC of 0.809 (95% CI, 0.690–0.905; sensitivity, 76.2%; specificity, 74.1%). Moreover, the RF classifier showed a good diagnostic performance for the N stage with an AUC of 0.746 (95% CI, 0.622–0.872; sensitivity, 79.3%; specificity, 72.2%) (Fig. 2).

## Discussion

This study indicated that the high-resolution T2WI–based radiomics machine learning model could not only differentiate pathological differentiation and T stage but also exhibited good diagnostic performance for N stage.

Recent studies have shown that radiomics is important in identifying tumor heterogeneity in several kinds of tumors [5–9], which may serve as a complementary tool for the preoperative tumor staging in rectal cancer [10–13]. The patients with rectal cancer required a comprehensive staging evaluation for guiding decisions regarding choice of treatment with an aim to avoid undertreatment and minimize overtreatment. Therefore, high-resolution T2WIs were used to explore the significance of MRI-based radiomics model in the preoperative diagnosis of rectal cancer in the present study.

Previous studies have shown by NCCN, degree of differentiation, T stage, and N stage are powerful prognostic factors for patients with rectal cancer [3]. Several studies showed a statistically significant correlation between the apparent diffusion coefficient value, derived from diffusion-weighted images, and tumor differentiation grade [16, 17]; however, some studies showed a contradictory result [18, 19]. In this study, radiomics and tumor differentiation grade showed a statistically significant correlation. The ROC curves of SVM classifier showed an AUC of 0.862 (test set), suggesting that the SVM model can be used to distinguish poorly differentiated lesions from highly/moderately differentiated lesions.

Although high-resolution MRI is recommended for the T staging of patients with rectal cancer, the accuracy of staging is still unsatisfactory. Some studies demonstrated differences in results that ranged from 44 to 100% [20, 21]. Stage T2 lesions could be differentiated from T3 lesions by identifying a smooth outer tumor border within the rectal wall, with no invasion into the fat surrounding the rectum. The difficulty in differentiating tumor infiltration from fibrosis, which is due to inflammation and blood vessel invasion, limited the ability to distinguish stage T2 tumors from early-stage T3 tumors [15]. In this study, the ROC curves of MLP classifier showed an AUC of 0.809 (test set), suggesting that the MRI-based radiomics model can be used to distinguish T3–4 lesions from T1–2 lesions. These results could be explained by the fact that higher T-stage tumors showed greater heterogeneity of cell morphology and histology, higher cell density, and smaller interstitium.

Accurate preoperative diagnosis of lymph node metastasis is another important factor for treatment selection. Although the accuracy of T staging is considerably high, the prediction of N staging remains difficult [22]. Using morphological criteria only does not improve the prediction accuracy of lymph node metastasis in rectal cancer [10]. This limitation is aggravated by the lack of consensus on appropriate criteria to assess lymph node involvement [20]. The reported accuracy of routine MRI for lymph node staging varied widely, ranging from 43 to 85% [23], suggesting that the MRI criteria for detecting lymph node metastasis are not reliable. However, the ROC curves of RF classifier showed an AUC of 0.746 (test set), which was partially consistent with the results of Huang’s study [24]. The study found radiomics signatures and other risk factors could conveniently facilitate the individualized preoperative prediction of lymph node metastasis in patients with CRC. Therefore, the RF model might reflect the aggressiveness of particular tumor tissue.

This study had several limitations. First, it was a retrospective study prone to selection bias, and the exclusion of patients with distant metastases limited its application. Hence, more patients should be included to validate the results. Second, due to the relatively small sample size, some lesions were nonuniformly distributed. Further studies are needed to broaden the application of radiomics for these lesions. Finally, radiomics is a recent imaging modality; the MRI scanning parameters and machine learning models are not yet standardized. Large prospective multicenter trials are necessary to fully evaluate the role of radiomics in the pathological features of rectal cancer.

## Conclusions

In conclusion, this study demonstrated that the high-resolution T2WI–based radiomics showed good classification performance related to tumor pathological features in patients with rectal cancer. Thus, radiomics may serve as a good alternative for evaluating the pathological features of rectal cancer and can add a further dimension to the predictive power of imaging.

## Data Availability

Not applicable

## Abbreviations

ADC: Apparent diffusion coefficient
AUC: Area under the curve
CRM: Circumferential resection margin
DWI: Diffusion-weighted imaging
FOV: Field of view
LASSO: Least absolute shrinkage and selection operator
MRI: Magnetic resonance imaging
NCCN: National comprehensive cancer network
RC: Rectal cancer
ROC: Receiver operating characteristic
TME: Total mesorectal excision
TR/TE: Repetition time/echo time
VOI: Volume of interest

## References

[1] Ferlay J, Soerjomataram I, Dikshit R, Eser S, Mathers C, Rebelo M (2015). Cancer incidence and mortality worldwide: sources, methods and major patterns in GLOBOCAN 2012. Int J Cancer.

[2] Chen W, Zheng R, Baade PD, Zhang S, Zeng H, Bray F (2016). Cancer statistics in China, 2015. CA Cancer J Clin.

[3] Benson AB, Venook AP, Al-Hawary MM, Cederquist L, Chen YJ, Ciombor KK (2018). Rectal Cancer, version 2.2018, NCCN clinical practice guidelines in oncology. J Natl Compr Cancer Netw.

[4] Lee YC, Hsieh CC, Chuang JP (2013). Prognostic significance of partial tumor regression after preoperative chemoradiotherapy for rectal cancer: a meta-analysis. Dis Colon Rectum.

[5] Gillies RJ, Kinahan PE, Hricak H (2016). Radiomics: images are more than pictures, They Are Data. Radiology.

[6] Lubner MG, Stabo N, Abel EJ, Del Rio AM, Pickhardt PJ (2016). CT textural analysis of large primary renal cell carcinomas: pretreatment tumor heterogeneity correlates with histologic findings and clinical outcomes. AJR Am J Roentgenol.

[7] Sidhu HS, Benigno S, Ganeshan B, Dikaios N, Johnston EW, Allen C (2017). Textural analysis of multiparametric MRI detects transition zone prostate cancer. Eur Radiol.

[8] Vargas HA, Veeraraghavan H, Micco M, Nougaret S, Lakhman Y, Meier AA (2017). A novel representation of inter-site tumour heterogeneity from pre-treatment computed tomography textures classifies ovarian cancers by clinical outcome. Eur Radiol.

[9] Ueno Y, Forghani B, Forghani R, Dohan A, Zeng XZ, Chamming's F (2017). Endometrial carcinoma: MR imaging-based texture model for preoperative risk stratification-a preliminary analysis. Radiology..

[10] Grone J, Loch FN, Taupitz M, Schmidt C, Kreis ME (2018). Accuracy of various lymph node staging criteria in rectal Cancer with magnetic resonance imaging. J Gastrointest Surg.

[11] Balyasnikova S, Read J, Wotherspoon A, Rasheed S, Tekkis P, Tait D (2017). Diagnostic accuracy of high-resolution MRI as a method to predict potentially safe endoscopic and surgical planes in patients with early rectal cancer. BMJ Open Gastroenterol.

[12] Sun Y, Hu P, Wang J, Shen L, Xia F, Qing G, et al. Radiomic features of pretreatment MRI could identify T stage in patients with rectal cancer: preliminary findings. J Magn Reson Imaging. 2018. 10.1002/jmri.25969 29437279

[13] Horvat N, Veeraraghavan H, Khan M, Blazic I, Zheng J, Capanu M (2018). MR imaging of rectal Cancer: Radiomics analysis to assess treatment response after Neoadjuvant therapy. Radiology..

[14] Mahul BA, Stephen E, Frederick LG (2016). AJCC cancer staging manual.

[15] Nougaret S, Reinhold C, Mikhael HW, Rouanet P, Bibeau F, Brown G (2013). The use of MR imaging in treatment planning for patients with rectal carcinoma: have you checked the "DISTANCE"?. Radiology..

[16] Cho EY, Kim SH, Yoon JH, Lee Y, Lim YJ, Kim SJ (2013). Apparent diffusion coefficient for discriminating metastatic from non-metastatic lymph nodes in primary rectal cancer. Eur J Radiol.

[17] Curvo-Semedo L, Lambregts DM, Maas M, Beets GL, Caseiro-Alves F, Beets-Tan RG (2012). Diffusion-weighted MRI in rectal cancer: apparent diffusion coefficient as a potential noninvasive marker of tumor aggressiveness. J Magn Reson Imaging.

[18] Sun Y, Tong T, Cai S, Bi R, Xin C, Gu Y (2014). Apparent diffusion coefficient (ADC) value: a potential imaging biomarker that reflects the biological features of rectal cancer. PLoS One.

[19] Tang C, Lin MB, Xu JL, Zhang LH, Zuo XM, Zhang ZS (2018). Are ADC values of readout-segmented echo-planar diffusion-weighted imaging (RESOLVE) correlated with pathological prognostic factors in rectal adenocarcinoma?. World J Surg Oncol.

[20] Al-Sukhni E, Milot L, Fruitman M, Beyene J, Victor JC, Schmocker S (2012). Diagnostic accuracy of MRI for assessment of T category, lymph node metastases, and circumferential resection margin involvement in patients with rectal cancer: a systematic review and meta-analysis. Ann Surg Oncol.

[21] Dewhurst C, Rosen MP, Blake MA, Baker ME, Cash BD, Fidler JL (2012). ACR appropriateness criteria pretreatment staging of colorectal cancer. J Am Coll Radiol.

[22] Tezcan D, Turkvatan A, Turkoglu MA, Bostanci EB, Sakaogulllari Z (2013). Preoperative staging of colorectal cancer: accuracy of single portal venous phase multidetector computed tomography. Clin Imaging.

[23] Bipat S, Glas AS, Slors FJ, Zwinderman AH, Bossuyt PM, Stoker J (2004). Rectal cancer: local staging and assessment of lymph node involvement with endoluminal US, CT, and MR imaging--a meta-analysis. Radiology..

[24] Huang YQ, Liang CH, He L, Tian J, Liang CS, Chen X (2016). Development and validation of a Radiomics Nomogram for preoperative prediction of lymph node metastasis in colorectal Cancer. J Clin Oncol.

